# Dynamic instability of genomic methylation patterns in pluripotent stem cells

**DOI:** 10.1186/1756-8935-3-17

**Published:** 2010-09-24

**Authors:** Steen KT Ooi, Daniel Wolf, Odelya Hartung, Suneet Agarwal, George Q Daley, Stephen P Goff, Timothy H Bestor

**Affiliations:** 1 Department of Genetics and Development, Columbia University, New York, USA; 2 HHMI and Department of Biochemistry and Molecular Biophysics, College of Physicians and Surgeons of Columbia University, New York, USA; 3 Stem Cell Program, Children's Hospital Boston, Boston, MA 02115, USA; 4 Stem Cell Transplantation Program, Division of Pediatric Hematology/Oncology, Manton Center for Orphan Disease Research, Howard Hughes Medical Institute, Children's Hospital Boston and Dana Farber Cancer Institute; Harvard Stem Cell Institute, Boston, MA 02115, USA; 5 Division of Hematology, Brigham and Women's Hospital, Boston, MA 02115, USA; 6 Department of Biological Chemistry and Molecular Pharmacology, Harvard Medical School, Boston, MA 02115, USA; 7 UCL Cancer Institute, Paul O'Gorman Building, University College London, London, WC1E 6BT, UK

## Abstract

**Background:**

Genomic methylation patterns are established during gametogenesis, and perpetuated in somatic cells by faithful maintenance methylation. There have been previous indications that genomic methylation patterns may be less stable in embryonic stem (ES) cells than in differentiated somatic cells, but it is not known whether different mechanisms of *de novo *and maintenance methylation operate in pluripotent stem cells compared with differentiating somatic cells.

**Results:**

In this paper, we show that ablation of the DNA methyltransferase regulator DNMT3L (DNA methyltransferase 3-like) in mouse ES cells renders them essentially incapable of *de novo *methylation of newly integrated retroviral DNA. We also show that ES cells lacking DNMT3L lose DNA methylation over time in culture, suggesting that DNA methylation in ES cells is the result of dynamic loss and gain of DNA methylation. We found that wild-type female ES cells lose DNA methylation at a much faster rate than do male ES cells; this defect could not be attributed to sex-specific differences in expression of DNMT3L or of any DNA methyltransferase. We also found that human ES and induced pluripotent stem cell lines showed marked but variable loss of methylation that could not be attributed to sex chromosome constitution or time in culture.

**Conclusions:**

These data indicate that DNA methylation in pluripotent stem cells is much more dynamic and error-prone than is maintenance methylation in differentiated cells. DNA methylation requires DNMT3L in stem cells, but DNMT3L is not expressed in differentiating somatic cells. Error-prone maintenance methylation will introduce unpredictable phenotypic variation into clonal populations of pluripotent stem cells, and this variation is likely to be much more pronounced in cultured female cells. This epigenetic variability has obvious negative implications for the clinical applications of stem cells.

## Background

*De novo *DNA methylation occurs primarily in non-dividing germ cells in a sexually dimorphic manner [1]. A key regulator of *de novo *methylation is the DNA methylation cofactor/adaptor DNMT3L (DNA methyltransferase 3-like). Genetic studies show that DNMT3L is required for the establishment of genomic imprints in growing oocytes [2] and for *de novo *methylation at retrotransposons in prospermatogonia [3]. Although DNMT3L possesses the structural folds present in all catalytically active mammalian DNA methyltransferases [4], it lacks the functional domains required for catalytic activity, and is unable on its own to cause DNA methylation [5]. Biochemical studies have demonstrated that DNMT3L can function as a regulator of the DNA methyltransferases DNMT3A and DNMT3B [6]. DNMT3L is not expressed in differentiated somatic cells but is expressed in embryonic stem (ES) cells, which are known to be highly active in DNA methylation [7,8]. We previously showed that DNMT3L forms a complex with DNMT3A2 and DNMT3B, and that this complex specifically binds to nucleosomes that are unmethylated at lysine 4 of histone H3 (H3K4) [4]. Biochemical studies revealed that DNMT3L interacts via the N-terminal cysteine-rich region with the N terminal tail of histone H3, and that this interaction is abolished by di- or trimethylation of H3K4. This resulted in the postulation of the DNMT3L histone recognition hypothesis, which states that recognition of DNA methylation target sequences is dependent on the ability of DNMT3L to bind the histone H3 N-terminus and that regulation of H3K4 methylation plays a role in targeted *de novo *DNA methylation. It is interesting to note that genomewide analysis of DNA methylation and H3K4 methylation, particularly di- and tri-methylation, reveals a mutually exclusive distribution [9], supporting the notion that H3K4 methylation protects promoter regions from *de novo *methylation.

Maintenance methylation is very stable in differentiated/somatic cells, and DNA that is methylated in predetermined patterns maintains this methylation pattern for >80 cell divisions in transfected cells [10]. This stability is a consequence of recognition of hemimethylated DNA after DNA replication by DNMT1 and the regulatory factor UHRF1 (ubiquitin-like, containing PHD and RING finger domains 1) [11]. Both DNMT1 and UHRF1 bind to hemimethylated CpG dinucleotides, and deficiency in either factor results in genomewide demethylation and embryonic lethality [12-14]. Additional mechanisms are likely to be involved in the correct recruitment of both DNMT1 and UHRF1. The observation that UHRF1 is able to bind to histone H3 that is di- or trimethylated at lysine 9 [15] implies the involvement of other chromatin factors.

Mitotic inheritance of genomic methylation patterns has been reported to be less faithful in ES cells than in differentiated somatic cells. A study of imprinted loci by Dean *et al*. [16] and Humphreys *et al*. [17] reported that methylation imprints are gained and lost at high rates in clonal populations of ES cells, although the mechanism of this was not apparent. Zvetkova *et al*. [18] reported spontaneous loss of methylation at imprinted and repeat sequences specifically in female ES cells; this was attributed to lower levels of DNMT3A/DNMT3B in XX cells.

We report here that mouse ES cells that lack DNMT3L lose methylation during culture, unlike non-stem cells, which maintain methylation patterns in the absence of DNMT3L. Loss of DNA methylation is much more rapid in female than in male mutant ES cells, even though levels of DNA methyltransferases and DNMT3L are the same in male and female ES cells. We also found that human ES and induced pluripotent stem (iPS) cells tend to lose DNA methylation spontaneously in a process that is independent of sex and passage number. Whereas maintenance methylation in non-stem cells is mediated by the faithful copying of methylation patterns at S phase, stem cell-specific maintenance of genomic methylation patterns involves dynamic demethylation and *de novo *methylation, which leads to heterogeneous methylation within clonal cell populations. This instability has the potential to cause dysregulation of imprinted genes and other gene expression abnormalities. Epigenetic instability is likely to introduce unpredictable phenotypic variation into clonal populations of ES and iPS cells, and the effect will be more severe when the cells are female.

## Results

**Figure 1:**
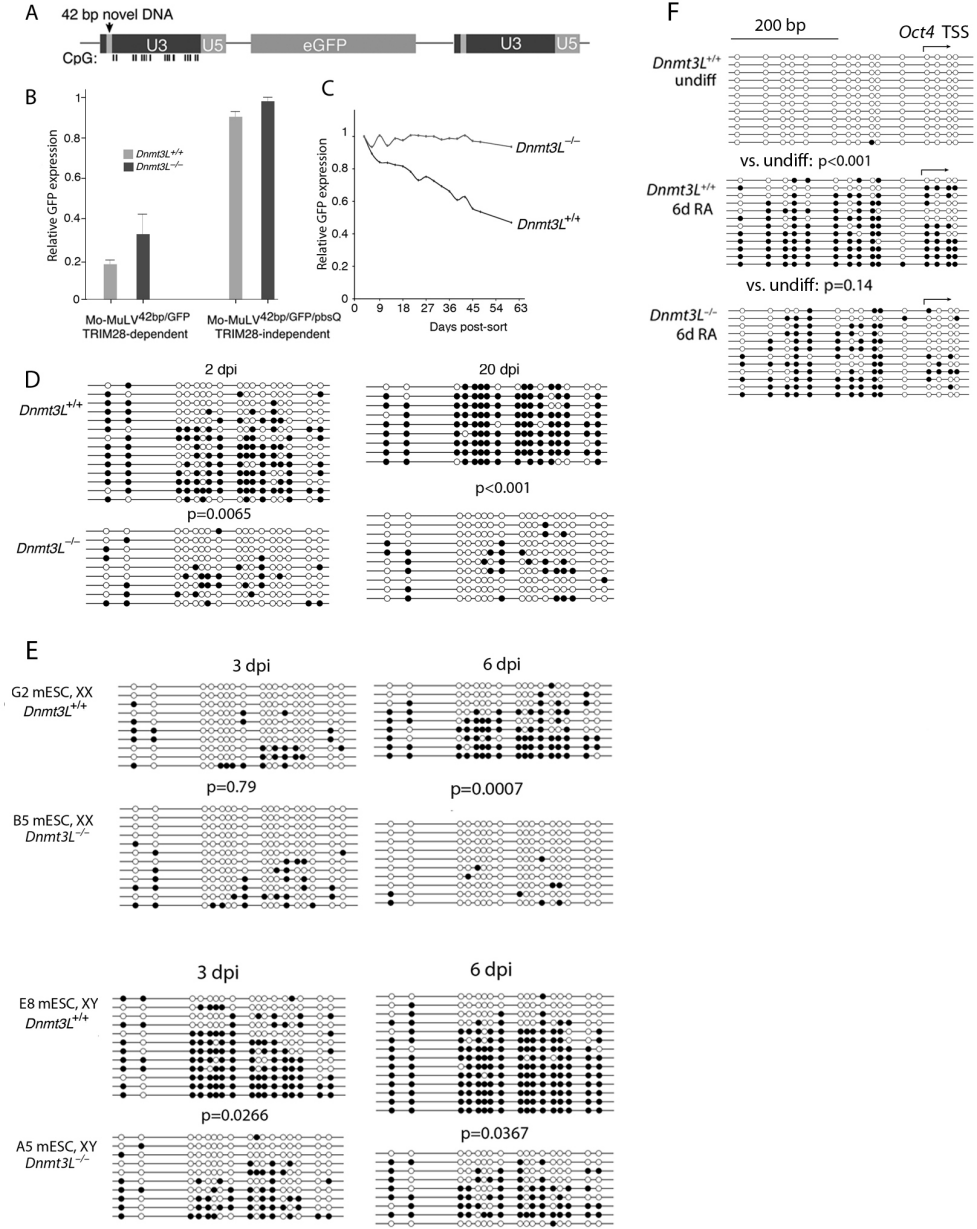
***De novo *methylation of proviral DNA requires DNMT3L (DNA methyltransferase 3-like)**. **(a) **Retrovirus reporter construct with long terminal repeats (LTRs) modified to allow identification of reporter provirus against background of endogenous retroviruses. **(b) **Replacement of primer binding site relieved TRIM28-ZFP809 mediated silencing, and the primer binding site was changed to be complementary to glutamine (Q) transfer (t)RNA. **(c) **Embryonic stem (ES) cells lacking DNMT3L were unable to silence the retrovirus shown in **(a)**. Green fluorescent protein (GFP)-expressing cells were isolated by flow sorting 3 days post-infection (dpi), and GFP expression was monitored by fluorescence-activated cell sorting over the time period indicated. **(d) **Lack of retrovirus silencing accompanied lack of LTR methylation. **(e) **Wild-type female ES cells were inefficient in *de novo *methylation, and methylation defect in female ES cells was seen in wild-type cells but was more severe in *Dnmt3L^-/- ^*ES cells. **(f) ***De novo *methylation of *Oct4 *5' region was not affected by loss of DNMT3L. All *P *values were obtained by the non-parametric two-tailed Mann-Whitney test.

Previous studies have demonstrated a requirement for DNMT3A and DNMT3B in the establishment of methylation in newly integrated Moloney murine leukemia virus (Mo-MLV) in ES cells [19]. We determined whether the regulatory factor DNMT3L was also required for this process. To allow the specific detection of the newly integrated retrovirus from the many copies endogenous to the mouse genome, we generated a Mo-MLV carrying an arbitrary 42 bp insertion within the U3 region of the long terminal repeat (LTR), (Mo-MLV^42bp/GFP^) (Figure 1a). The retroviral construct was further rendered resistant to TRIM28-ZFP809 mediated-restriction [19,20] by replacement of the transfer (t)RNA^Pro ^primer binding site (PBS) with a tRNA^Gln ^PBS. This made the provirus largely resistant to TRIM28-ZFP809-dependent transcriptional silencing (Figure 1b). However, infection of wild-type ES cells resulted in the gradual silencing of proviral expression during passage. Ablation of DNMT3L largely prevented the passage-dependent silencing of retroviral transcription that occurs in the presence of DNMT3L, and this lack of silencing activity was associated with a failure to methylate the LTRs of the Mo-MLV^42bp/GFP ^retrovirus (Figure 1c).

It was surprising to find that female (XX) ES cells were much less proficient at provirus methylation than were male (XY) ES cells (Figure 1d), both in the presence and absence of DNMT3L. Also surprising was the DNMT3L-independent *de novo *methylation of the *Oct4 *promoter (Figure 1e), which normally occurs when ES cells are induced to differentiate [20]. However, there are both DNMT3L-dependent and -independent *de novo *methylation events in germ cells [2,3].

**Figure 2:**
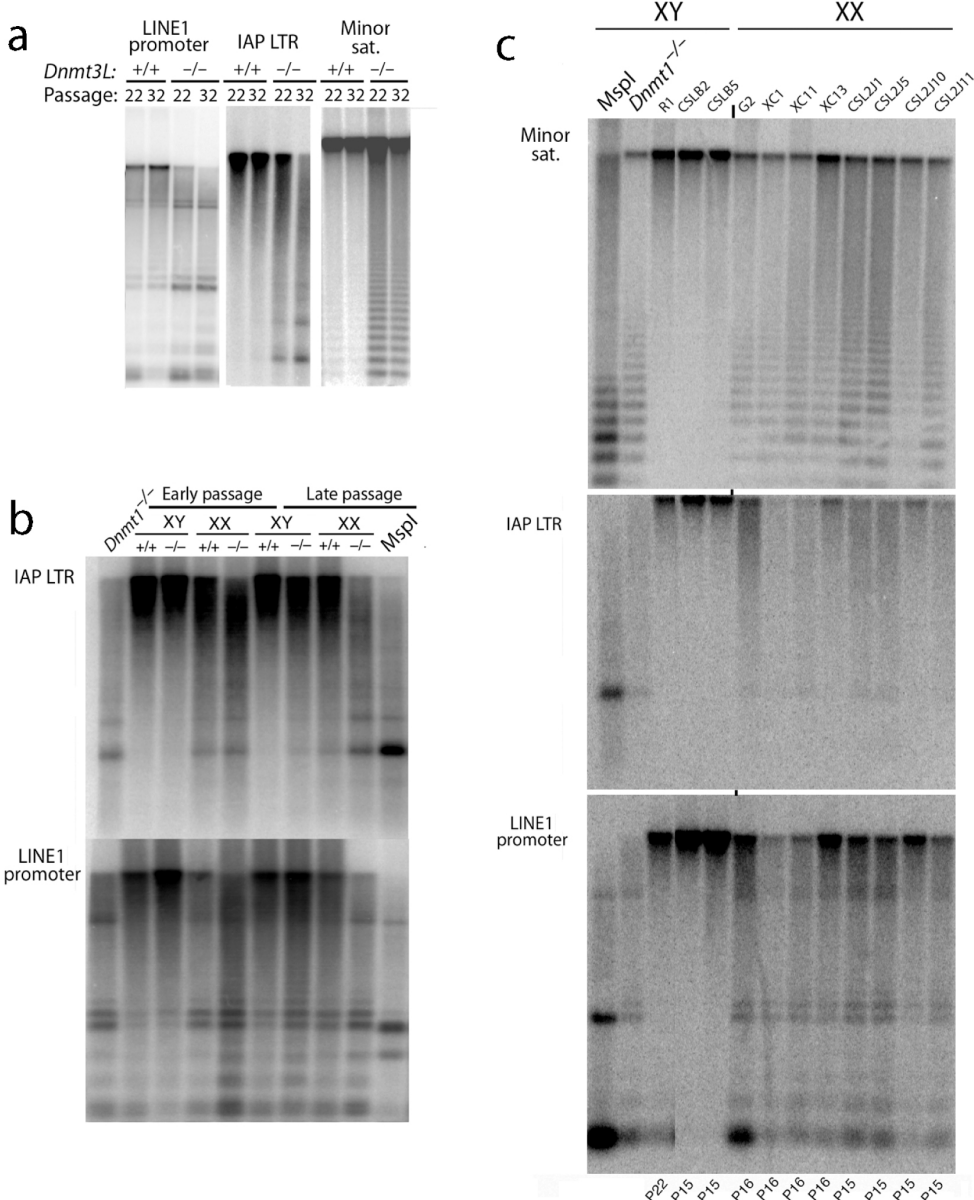
**Time- and sex-dependent loss of DNA methylation in absence of DNMT3L (DNA methyltransferase 3-like)**. All embryonic stem (ES) cell clones analyzed had typical ES cell morphology and comparable growth rates (data not shown). **(a) **Culture of *Dnmt3L^-/- ^*ES cells caused a loss of DNA methylation from multiple repeat-sequence classes. LINE1 promoters, Intracisternal A Particle (IAP) long terminal repeats (LTRs) and minor satellite are shown. **(b) **Loss of methylation in XX *Dnmt3L^-/- ^*ES cells was more rapid than in XY *Dnmt3L^-/- ^*ES cells. Late passage XX *Dnmt3L^-/- ^*ES cells showed an extent of demethylation that was similar to that of ES cells null for *Dnmt1*. *Dnmt3L *genotype is indicated by '+/+' and '-/-' at the top of each lane. **(c) **Comparison of DNA methylation in XY and XX ES cells. Three normal XY ES cell lines were compared with eight normal XX ES cell lines. Demethylation was more pronounced in all of the XX lines compared with XY cells.

As shown in Figure 2a, DNMT3L is required for the maintenance of genomic methylation patterns in ES cells. DNMT3L is not required for maintenance methylation in somatic cells, as it is not expressed in differentiated somatic cells, and ablation of DNMT3L results in normal DNA methylation and normal mouse development [1]. Both *Dnmt3L^-/- ^*and *Dnmt3^+/+ ^*XX ES cells lost methylation at much higher rates than did XY ES cells (Figure 2b). All eight independent XX ES cell lines tested showed much greater loss of methylation than did the three XY cell lines tested (Figure 2c).

**Figure 3:**
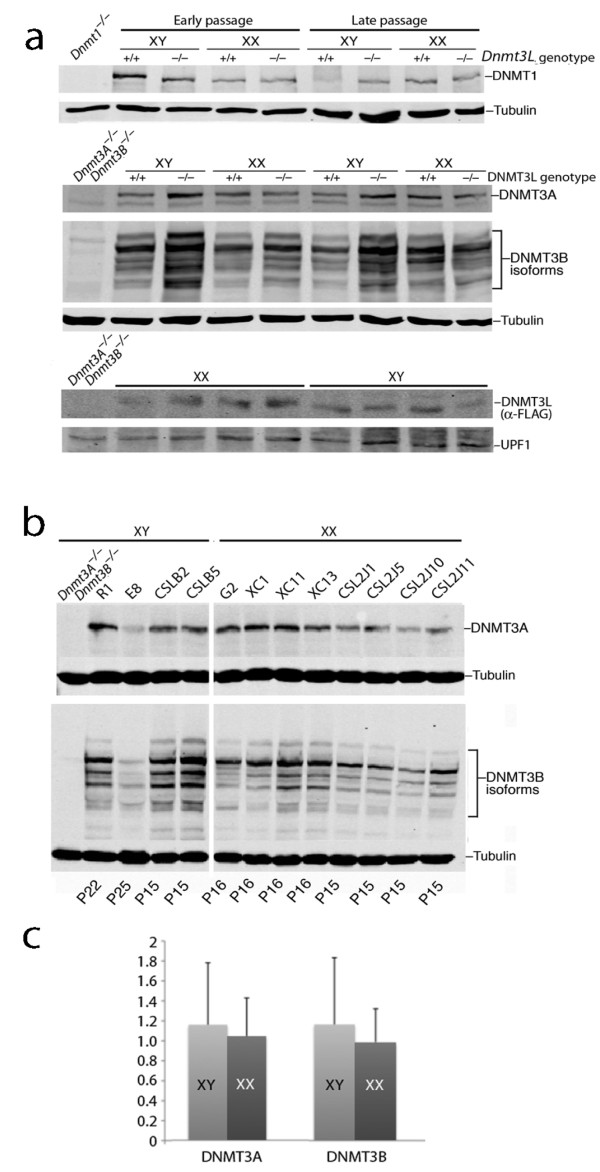
**Normal expression of DNA methyltransferase (DNMT)1, DNMT3A, DNMT3B and DNA methyltransferase 3-like (DNMT3L) in XY and XX embryonic stem (ES) cells**. (**a) **All three DNMTs and DNMT3L were expressed at similar levels regardless of passage history or sex chromosome substitution. **(b) **Comparison of a panel of eight XX and three XY ES cell lines showed similar expression of DNMT3A and DNMT3B. **(c) **Quantification of DNMT3A and DNMT3B expression. Signal intensities were measured by an imaging system as described in the text, and normalized against tubulin. The data showed that that XY and XX ES cells had very similar levels of DNMT3A and DNMT3B. Error bars indicate one standard deviation.

It had been previously reported that wild-type XX ES cells lose methylation with continued passage in culture, with reduced expression of both DNMT3A and DNMT3B in XX cells being reported as the cause [18]. However, our quantitative immunoblot analysis (Odyssey^® ^Infrared Imaging System; Li-COR Biotechnology, Lincoln, NB, USA) showed that levels of DNMT1, DNMT3A, DNMT3B and DNMT3L were very similar in XX and XY ES cells (Figure 3a-c), and that their levels were very similar in early and late passage ES cells (Figure 3a, b). The numerous isoforms of DNMT3B that arise via alternative splicing were also very similar in XX and XY ES cells (Figure 3a, b). These data indicate that the cause of XX-specific demethylation is not reduced expression of DNA methyltransferases.

**Figure 4:**
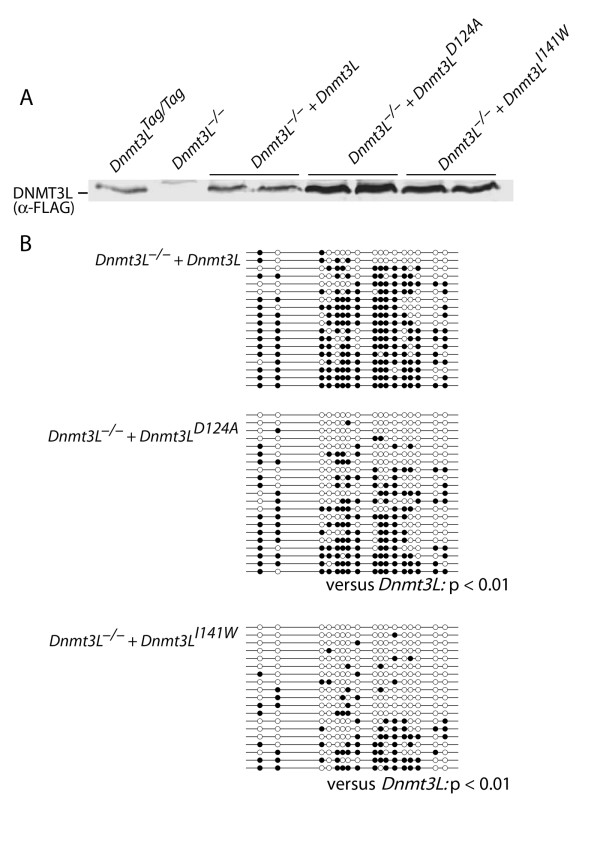
**DNMT3L (DNA methyltransferase 3-like) that is unable to bind unmethylated H3K4 is deficient in rescue of the *de novo *methylation defect in *Dnmt3L^-/- ^*embryonic stem (ES) cells**. The D90A and I107W substitutions were previously shown to prevent binding of human DNMT3L to histone H3 peptide unmethylated at lysine position 4 (H3K4) [4]. The equivalent residues in mouse DNMT3L are D124A and I141W, respectively. **(a) **The mutant proteins were stable and were expressed at higher levels than the wild-type protein; two independent stable transfectants are shown for each. **(b) **Failure of mutant DNMT3L to rescue the *de novo *methylation defect. The long terminal repeat (LTR) of the retrovirus shown in Figure 1(a) was tested for *de novo *methylation at 12 days post-infection. These data demonstrate that *de novo *methylation in ES cells requires the interaction of DNMT3L and unmethylated H3K4.

The finding that DNMT3L is required for *de novo *methylation of retroviral DNA allowed us to test the importance of the interaction of DNMT3L with unmethylated H3K4. Mutations that caused amino acid substitutions at positions necessary for the interaction of DNMT3L and histone H3 [4] were introduced into DNMT3L expression constructs and stably expressed in ES cells. The recombinant proteins were stable and were expressed at levels equal to or greater than that of endogenous DNMT3L or of transfected wild-type DNMT3L (Figure 4a). Disruption of the DNMT3L-H3K4 interaction caused a partial reduction of methylation in the case of the D124A substitution and a severe reduction in *de novo *methylation in the case of the I141W mutation (Figure 4b). These data indicate that the bulk of *de novo *methylation mediated by DNMT3L is probably regulated by the association of unmethylated DNA with nucleosomes that are enriched in unmethylated H3K4.

**Figure 5:**
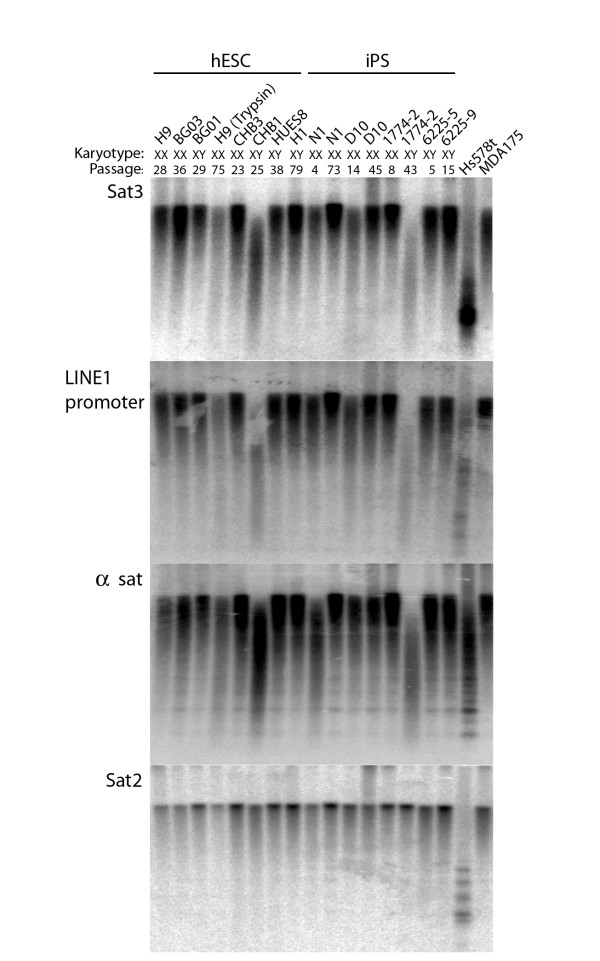
**Instability of genomic methylation patterns in human embryonic stem (ES) and induced pluripotent stem (iPS) cells**. DNA was analyzed for demethylation at the sequences named on the left. Controls were the human mammary carcinoma cell line Hs578T, which has a severely demethylated genome, and the MDA175 cell line, which is not detectably demethylated. Note that all human ES and iPS cells showed demethylation of a satellite relative to MDA175 and that trypsin-passaged H9 and CHB1 human ES and D10 cells and high-passage 1774-2 iPS cells showed demethylation of LINE1 promoters and satellite 3 DNA. All human ES and iPS lines were maintained under the same cell culture conditions, without the use of trypsin except in the case of the indicated H9 cell line, for five or more passages.

The finding that mouse ES cells display sex-specific epigenetic instability suggested that human ES and iPS cells might show a similar instability. Human ES and iPS lines were tested for loss of methylation at LINE1 transposon promoters, α-satellite DNA, satellite 2 DNA from chromosomes 1 and 16, and satellite 3 DNA from chromosome 9. All human ES and iPS lines showed loss of methylation at α-satellite DNA, and two human ES lines and two iPS lines showed marked demethylation of satellite 3 and LINE1 promoters; satellite 2 was not affected in any cell line (Figure 5). Demethylation was not strongly associated with passage history or sex chromosome constitution, and the N1 and D10 iPS lines showed greater demethylation at early than at later passages. These data indicate that human ES and iPS cells are subject to marked epigenetic instability under conditions of normal *ex vivo *propagation.

## Discussion

ES and embryonic carcinoma cells are known for their ability to potently restrict retroviral expression [7,8], which involves two phases. The initial phase, which immediately follows retroviral integration, depends on the interaction of retroviral DNA sequences with host restriction factors, which include TRIM28 [21] and ZFP809 [22]. Maintenance of this repression is subsequently thought to rely on epigenetic mechanisms, primarily DNA methylation. Clonal studies in ES cells using murine stem cell virus (MSCV) transduction followed by knockdown of DNMT3A and/or DNMT3B showed that maintenance of DNA methylation is important for stable proviral silencing [23], and 5-azacytidine-induced demethylation of previously methylated and silent MSCV provirus resulted in their reactivation [24]. Both these observations suggest that DNA methylation is necessary to enforce provirus silencing. By starting with a population of ES cells in which integrated provirus has escaped the initial silencing system by virtue of replacement of the primer binding site to prevent binding of ZFP809, we investigated the role of DNMT3L-mediated DNA methylation acquisition in the gradual silencing of active retrovirus. We found that the ability to silence over time is dependent on the ability to acquire methylation at proviral LTRs.

Whereas DNMT3L was found to be necessary for *de novo *methylation of newly integrated proviral DNA, it was dispensable for *de novo *methylation at a promoter after induction of differentiation. *In vitro *differentiation of ES cells is known to coincide with *de novo *methylation at over 300 CpG-poor regions that are in proximity to gene promoters [25]. This is evidence of DNMT3L-independent *de novo *methylation, which had been previously reported [2,3]. It is not clear whether the low density of DNA methylation actually represses transcription or whether the *de novo *methylation of the CpG-poor *Oct4 *promoter is actually involved in *Oct4 *regulation.

Previous studies in mouse ES and iPS cells have reported that the presence of two X chromosomes causes genomewide hypomethylation [18,26]. Our quantitative studies examined the expression levels of DNMT1, DNMT3A and DNMT3B in XX and XY ES cells, and revealed that the increased rate of loss of DNA methylation in XX versus XY ES cells cannot be attributed to reduced amounts of DNA methyltransferase proteins. It is instead consistent with some inherent difference between XX and XY ES cells, which affects DNMT recruitment and general regulation of DNA methylation. XX cells may have a higher rate of loss of methylation or a lower rate of remethylation, or both. The observation that DNMT3L deficiency results in hypomethylation at retrotransposons and minor satellite sequences is also in contrast to previously published results, which claimed that DNMT3L was dispensable for their methylation [27]. Given that we observed a passage-dependent effect on the ability to maintain methylation in the absence of DNMT3L (Figure 2a, b), we propose that this discrepancy might be attributable to lower passage numbers of the DNMT3L-deficient ES cells used in the earlier analysis.

Two previous studies reported that the active methyltransferases DNMT3A and DNMT3B were required for methylation content to be maintained at normal levels [28,29]. Our study is the first to demonstrate that the catalytically inactive adaptor DNMT3L is required for normal DNA methylation in pluripotent stem cells. DNMT3L is not expressed in differentiated somatic cells, yet unlike *Dnmt3L*-deficient ES cells, they are able to maintain genomic methylation patterns with high fidelity. These findings indicate that methylation patterns in ES cells are the product of the dynamic gain and loss of DNA methylation, rather than passive clonal inheritance as occurs in differentiated cells. This places a higher load on non-maintenance methylation-based mechanisms, which involve DNMT3 family members. We speculate that there are at least two possible explanations why maintenance methylation-based mechanisms (that is, those involving DNMT1 and UHRF1) are less effective in ES cells. First, ES cells contain combinations of histone modifications not observed in differentiated somatic cells, which could adversely affect recruitment of DNMTs and other factors involved in maintenance methylation. Among these is bivalent methylation of H3K4 and H3K27, two methylation markers that are usually mutually exclusive [30]. Although it has been shown that UHRF1 binds to di- and trimethylated H3K9 [15], the consequences of H3K27 methylation and of other ES cell-specific patterns of chromatin modifications are unknown but are likely to be responsible for some of the epigenetic instability that occurs in pluripotent stem cells. Second, 5-hydroxymethyl cytosine (hm^5^C) is present in DNA of ES cells, [31] and structural models indicate that UHRF1 cannot bind to hm^5^C [32]. If hm^5^C occurs within CpG dinucleotides, this could lead to inefficient maintenance methylation. However, the sequence contexts in which hm^5^C occurs *in vivo *are not known, and the role of this modified base in maintenance methylation is unclear.

## Conclusions

The most significant feature of unstable genomic methylation patterns in pluripotent stem cells may be the introduction of stochastic phenotypic variation into clonal cell populations, particularly with regard to genome destabilization, selection of cells that have increased expression of genes that stimulate cell growth, and the unpredictable gain and loss of imprinted gene expression. It should be noted that cultured ES cells are derived from cell types that exist only transiently *in vivo*. Selective pressures for high genetic [33] or epigenetic stability are therefore low *in vivo*. The forced *ex vivo *propagation of ES cells for a far greater number of cell divisions than are undergone by their *in vivo *counterparts renders cultured stem cells - both ES and iPS cells -vulnerable to increased genetic and epigenetic instability.

## Methods

### Cell culture and sample preparation

DNMT3L-deficient ES cells were derived from crosses between *Dnmt3L^+/- ^*animals [2] using a previously described protocol [34]. Dnmt1^-/- ^and *Dnmt3a^-/-^;Dnmt3b^-/- ^*ES cells (generously provided by E. Li, Novartis, MA, USA) have also been described previously [19]. Mouse ES cells were cultured on gelatinized tissue culture plates in ES cell media (Dulbecco modified Eagle medium (DMEM) supplemented with 15% fetal bovine serum, 100 IU/mL penicillin, 100mg/mL streptomycin, 2 mmol/L L-glutamine, MEM non-essential amino acids, 0.12 mmol/L β-mercaptoethanol, and leukemia inhibitory factor (LIF) from the conditioned medium of LIF-secreting cells. Human ES and iPS cells were cultured on γ-irradiated (CF1-derived) mouse embryonic fibroblasts (GlobalStem Inc., Rockville, MD, USA) in standard human ES media (DMEM/F-12; Stem Cell Technologies Inc., Vancouver, BC, Canada) supplemented with 20% knockout serum, 1 mM L-glutamine and 100 μM MEM-nonessential amino acids (Invitrogen Corp., Carlsbad, CA, USA), 100 μM 2-mercaptoethanol (Sigma Chemical Co., St Louis, MO, USA) and 10ng/ml recombinant human bFGF (Invitrogen). Both mouse and human ES and iPS cell DNA was purified using a commercial kit (DNeasy^® ^Blood and Tissue Kit; Qiagen Inc., Valencia, CA, USA).

### Flow cytometry

For sorting experiments, green fluorescent protein-positive ES cells were purified on a cell sorter (FACSAria Cell Sorter; BD Biosciences, San Jose, CA, USA), and analyses performed on an automated cell analyzer (FACSCalibur Cell Analyzer; BD Biosciencs).

### Methylation analysis

Retroviral preparation and transduction was performed as described previously [19]. For analysis of Mo-MLV^40bp/GFP^, bisulfite conversion using the method described by Hajkova and colleagues [35] was used. For *Oct4 *promoter analysis, DNA was converted using a commercial kit (EZ DNA Methylation Gold™Kit; Zymo Research Corp., Orange, CA, USA). Analysis and statistical comparison of bisulfite data was performed using QUMA software http://quma.cdb.riken.jp/[36]. For methylation-sensitive Southern blots, DNA was subjected to 2 rounds of digestion with either methylation-sensitive *HpaII *or the methylation-insensitive isoschizomer *MspI *(New England Biolabs Inc., Ipswich, MA, USA) to ensure complete digestion. Briefly, DNA was purified from cell pellets (fresh or frozen) using a commercial kit (DNeasy Kit; Qiagen) according to the manufacturer's protocol and quantified, before digesting using a 10-fold unit excess of enzyme. After digestion, DNA was precipitated with ethanol and digested a second time. Digestions were performed for between 4 and 6 hours. Digested DNA was resolved in 1% agarose gels, before being transferred onto a nylon membrane. After ultraviolet-induced cross-linking, membranes were incubated at 65°C with prehybridization solution (6 × saline sodium citrate buffer, 10 × Denhardt solution, 1% sodium dodecyl sulfate, 10% dextran sulfate). LINE1 and Intracisternal A Particle (IAP) probes were used as described previously [37]. The minor satellite probe used has also been described previously [38]. Probes were incubated with membranes overnight, before washing and exposure to phosphor screens (Phosphorimager; Molecular Dynamics, Sunnyvale, CA, USA).

### Statistical analysis

All statistical comparisons were carried out using the non-parametric two-tailed Mann-Whitney test.

### Antibodies

For western blotting, the antibodies used were: anti-DNMT1 rabbit polyclonal (pATH52) [37] 1: 800; anti-DNMT3A (SC-20703; Santa Cruz Biotechnologies, Santa Cruz, CA, USA) 1:100; anti-DNMT3B (SC-52922; Santa Cruz Biotechnologies) 1: 200; anti-tubulin mouse monoclonal (T6199; Sigma Chemical Co. St. Louis, MO, USA,); anti-UPF1 Rent1 (H300) (SC-48802; Santa Cruz Biotechnologies), anti-FLAG M2, mouse monoclonal (F3165; Sigma Chemical Co.) 1: 400. Horse radish-conjugated secondary antibodies were obtained from Sigma Chemical Co. IR-800 antibodies used for the Li-Cor detection system were obtained from Rockland Immunochemicals (Gilbertsville, PA, USA).

### Primer sequences

Primers used for bisulfite analysis were designed using MethPrimer [39]. The primer sets used are set out in Table 1

**Table 1:** Primer sets used in the experiments

Name	Sequence 5'→3'
Nested primers for bisulfite analysis of Mo-MLV^GFP ^long terminal repeat	

GFPF1	TTTTTTTATATATTATTATTTATTTTTTTT

GFPR1	ATCAATCACTCAAAAAAAACCCTC

GFPF2	TAGGGTTAAGAATAGATGGAATAGTTGA

Bisulfite analysis of *Oct4 *promoter	

Oct4F1	TGAGGAGTGGTTTTAGAAATAATTG

Oct4R1	AAACCAAATATCCAACCATAAAAAA

Oct4R2	CCAACCATAAAAAAAATAAACACC

Mo-MLV^42bp/GFP ^creation^1^	

Forward	TTATACTCCTCCACACACCATCACTCACTCTTTCTCAATCCA

Reverse	TGGATTGAGAAAGAGTGAGTGATGGTGTGTGGAGGAGTATAA

^1^Moloney murine leukemia virus (Mo-MLV)^42bp/GFP ^was created by inserting this synthetic oligonucleotide into the U3 region of the Mo-MLV long terminal repeat.

## Competing interests

The authors declare that they have no competing interests.

## Authors' contributions

SKTO, THB and DW contributed to the study design. SKTO, DW, OH and SA contributed to the experimental work. SKTO, THB, GD and SPG contributed to manuscript preparation. With the exception of DW, all authors read and approved the final manuscript.
